# Iguratimod attenuated fibrosis in systemic sclerosis via targeting early growth response 1 expression

**DOI:** 10.1186/s13075-023-03135-2

**Published:** 2023-08-18

**Authors:** Lichong Shen, Hanlin Yin, Li Sun, Zhiliang Zhang, Yuyang Jin, Shan Cao, Qiong Fu, Chaofan Fan, Chunde Bao, Liangjing Lu, Yifan Zhan, Xiaojiang Xu, Xiaoxiang Chen, Qingran Yan

**Affiliations:** 1 Department of Rheumatology, Ren Ji Hospital, Shanghai Jiao Tong University School of Medicine, Shanghai, 200001 China; 2 Department of Rheumatology and Immunology, The First Affiliated Hospital of Wenzhou Medical University, Wenzhou, 325000 China; 3 Department of Plastic Surgery, Ren Ji Hospital, Shanghai Jiao Tong University School of Medicine, Shanghai, 200001 China; 4 Department of Drug Discovery, Shanghai Huaota Biopharm, Shanghai, 201203 China; 5 Department of Pathology and Laboratory Medicine, Tulane University School of Medicine, New Orleans, LA USA; 6 Department of Rheumatology, Nantong First People’s Hospital, Affiliated Hospital 2 of Nantong Universuty, Nantong Hospital of Renji Hospital Affiliated to Shanghai Jiao Tong Universuty School of Medicine, Nantong, 226006 China

**Keywords:** Early growth response 1, Systemic sclerosis, Iguratimod, Anti-fibrotic, Xenograft

## Abstract

**Background:**

The early growth response 1 (EGR1) is a central transcription factor involved in systemic sclerosis (SSc) pathogenesis. Iguratimod is a synthesized anti-rheumatic disease-modifying drug, which shows drastic inhibition to EGR1 expression in B cells. This study is aiming to investigate the anti-fibrotic effect of iguratimod in SSc.

**Methods:**

EGR1 was detected by immunofluorescence staining real-time PCR or western blot. Iguratimod was applied in EGR1 overexpressed or knockdown human dermal fibroblast, bleomycin pre-treated mice, tight skin 1 mice, and SSc skin xenografts. RNA sequencing was performed in cultured fibroblast and xenografts to identify the iguratimod regulated genes.

**Results:**

EGR1 overexpressed predominantly in non-immune cells of SSc patients. Iguratimod reduced EGR1 expression in fibroblasts and neutralized changes of EGR1 response genes regulated by TGFβ. The extracellular matrix (ECM) production and activation of fibroblasts were attenuated by iguratimod while EGR1 overexpression reversed this effect of iguratimod. Iguratimod ameliorated the skin fibrosis induced by bleomycin and hypodermal fibrosis in TSK-1 mice. Decreasing in the collagen content as well as the density of EGR1 or TGFβ positive fibroblasts of skin xenografts from naïve SSc patients was observed after local treatment of iguratimod.

**Conclusion:**

Targeting EGR1 expression is a probable underlying mechanism for the anti-fibrotic effect of iguratimod.

**Supplementary Information:**

The online version contains supplementary material available at 10.1186/s13075-023-03135-2.

## Background

Systemic sclerosis (SSc) is an idiopathic connective tissue disease characterized by organ fibrosis, especially in the skin and lung [1]. It is proposed as one prototype of fibrotic disease [2, 3] Fibrosis leads to organ failure in the end-stage of the disease.

Early growth response-1 (EGR1) is a zinc-finger DNA-binding protein and belongs to the family of ligand-inducible early response genes (EGRs). EGR1 is a ubiquitous transcription factor deeply implicated both in the profibrotic and inflammation process. As a positive regulator of the profibrotic process, EGR1 constructs a positive feedback loop with transformation growth factor-β (TGF-β), the central cytokine for fibroblast activating and functioning [4]. TGF-β stimulates the expression of EGR1 through non-canonical c-Abl/p300 signaling [5, 6] and EGR1 binds the TGFB1 promoter and enhances the expression in turn [7]. In addition, EGR1 is sufficient to directly bind to collagen gene promoters to stimulate collagen synthesis [5, 8]. On the other hand, EGR1 is a key controller of macrophage inflammation [9] and peripheral maturity of B cells [10, 11].

For SSc patients, almost every well-known environmental pathogenic factor of SSc can stimulate the expression of EGR1, including reactive oxidants, hypoxia, and UV light [4]. In fact, EGR1, as well as EGR1-response genes, are found overexpressing in the skin lesions of diffuse SSc patients, which is proposed as an “EGR1 signature” of SSc [12]. Although EGR1 is proven to participate in the regulation of both fibroblast and immune cells’ function in numerous researches, the exact pathological process, which is regulated by EGR1 in the onset or progression of SSc still remains unclear.

So far, there is neither direct evidence supporting EGR1 as a therapeutic target in SSc nor EGR1 inhibitor available in clinical practice. While in basic research, a DNA enzyme targeting Egr1 was reported to ameliorate renal fibrosis in unilateral ureteral obstruction rat [13]. In addition, a peptide derived from endostatin [14] showed an anti-fibrotic effect in bleomycin-induced lung fibrosis by reducing Egr1 production in fibroblasts. For approved medications, only a peroxisome proliferator-activated receptor-γ agonist pioglitazone [15] improves TGF-β induced renal fibrosis with synchronized lowered EGR1 expression. However, this evidence is far from enough to demonstrate that EGR1 is the key mechanism for the reagents to relieve fibrosis.

In recent decades, a new synthesized anti-rheumatic drug, iguratimod, has emerged as a potential candidate that efficiently inhibits EGR1 expression. It has been approved for the treatment of rheumatoid arthritis (RA) in northeast Asia. We have recently identified EGR1 as one of the most significantly downregulated genes in B cells upon iguratimod treatment [16]. Therefore, we speculated that iguratimod might be suitable for repurposing to SSc. In this study, on top of the efficacy investigation of iguratimod in SSc models, we will try to interrogate the underlying mechanism of its anti-fibrotic effects.

## Materials and methods

### Patients

All the patients in this work fulfilled the classification criteria of SSc by ACR/EULAR in 2013 [17]. Written informed consent was obtained from all participants of this study. For EGR1 staining, skin biopsy samples from eight dcSSc patients and eight age and gender-matched healthy controls were collected. Demographic information of these patients is in Table S1. For skin xenograft, lesional skin tissues were obtained from three treatment naïve SSc patients whose mRSS (modified Rodnan skin score) > 10. Skin punch biopsies were divided into two pieces and transplanted subcutaneously into irradiated nude mice. Ten micrograms/liter iguratimod suspended in normal saline (with 10% DMSO) was injected around the skin graft at 100 µL/day every other day for 5 weeks.

### Mice and treatments

Male C57BL/6(B6) mice and nude mice were purchased by Shanghai SLAC Laboratory Animal Co., Ltd. Tight skin-1 (Tsk-1) mice were purchased from Jackson Laboratories (Bar Harbor, Maine, USA). Both strains were bred in a specific pathogen-free facility. Animal experiments were carried out according to institutionally approved protocols of the Animal Care and Use Committee of Shanghai Jiao Tong University, Shanghai, China.

### Bleomycin-induced skin fibrosis and treatment

Six-week-old female C57/bl6 mice were injected with bleomycin subcutaneously as previously described [18]. After a 3-week injection, the mice were given oral iguratimod (30 mg/kg/day) or topical 1% DMSO-dissolved iguratimod 50 µl twice a day for another 3 weeks, with continued bleomycin injection.

### Tsk-1 mice treatment

Six-week-old Tsk-1 mice were given 1% iguratimod in 100ul DMSO solution topically on a 1cm^2^ area of back twice a day. The treatment lasted for 6 weeks.

### Primary skin fibroblast isolation and treatment

Primary dermal fibroblasts were isolated from the foreskin of healthy donors, and passage 4 to 8 was used for experiments. The fresh foreskin tissue was digested with 0.1% dispase II overnight for removing the departed epidermis, then was treated the dermis with 0.5% collagenase (Serva, Germany). Fibroblasts were harvested and maintained in Dulbecco’s modified Eagle’s medium (DMEM) supplemented with 10% fetal bovine serum (FBS) (Gibco BRL, Grand Island, NY), 1% vitamin solutions, 2 mM L-glutamine, and 1% Penicillin–Streptomycin Solution in a humidified 5% CO2 atmosphere.

We used 10 ng/mL recombinant TGF-β (R&D Systems, Abingdon, UK) to stimulate fibroblast. Fibroblasts were simultaneously treated with different concentrations of iguratimod, which was kindly supplied by Simcere Pharmaceutical (Nanjing, China).

### Transcriptome profiling

Total RNA from cultured fibroblasts or xenografts was extracted by TRIzol (Invitrogen) reagent. For the library preparation, 2 µg total RNA was captured by NEBNext Oligo d(T)25 beads (NEB, USA), sheared to fragments of ~ 250 bp, and reverse transcribed by NEBNext RNA first and Strand Synthesis Module second (NEB, USA). The products were end-repaired, A-tailed, ligated to Illumina sequencing adapters, and amplified by PCR. The sequencing library was qualified by Qubit 2.0 (Life Technologies, USA) and Bioanalyzer 2100 (Agilent, Germany), then sequenced on Illumina Hiseq2500 with 150 bp paired-end sequencing, which was controlled by Hiseq Control Software (HCS).

Differential expression analysis of two groups was performed using DESeq2 R package (1.10.1). DESeq2 provides statistical routines for determining differential expression in digital gene expression data using a model based on the negative binomial distribution. The resulting *P* values were adjusted using Benjamini and Hochberg’s approach for controlling the false discovery rate. Gene mapping comparison between human and mouse was used to filter the contaminated mouse genes. Genes with an adjusted *P* value < 0.05 and absolute fold change larger than 1.5 were assigned as differentially expressed. Principle component analysis (PCA) was implemented with R package prcomp.

Heatmap was used to demonstrate the expression patterns of differentially expressed E1 response genes. Pathway analysis was performed using Ingenuity Pathway Analysis (IPA) software from the Qiagen system, to identify enriched canonical pathways and top upstream regulators of essential genes. Gene Set Enrichment Analysis (GSEA) was performed using software from Broad Institute searching for pathways whose genes are enriched at the top or bottom of the ranked gene list, more so than expected by chance alone.

### siRNA or plasmid transfection

EGR1-specific and non-target (n.t.) siRNA oligos were synthesized by GenePharma Co. Ltd. The siRNA oligonucleotide sequences were EGR1 siRNA sense 5′-CCAUGGACAACUACCCUAATT-3′ anti-sense 5′-UUAGGGUAGUUGUCCAUGGTT-3′ and non-targeting siRNA, sense 5′-UUCUCCGAACGUGUCACGUTT-3′, anti-sense 5′-ACGUGACACGUUCGGAGAATT-3′. The human dermal fibroblasts were transfected with 3 μg EGR1 siRNA or non-targeting siRNA using Lipofectamine RNAiMAX Transfection Reagent (Thermo Fischer, USA) according to the manufacturer’s protocol. Overexpression of EGR1 in dermal fibroblasts was induced by transfection with 1 μg pcDNA3.1 plasmid encoding the gene sequence of human EGR1 Open reading frame (Genscript USA). Healthy dermal fibroblasts transfected with an equal amount of empty pcDNA3.1 plasmid as controls. Transfection was performed using the 4D-Nucleofector (Lonza) following established protocols.

### Quantitative real-time PCR

Total RNA from cultured cells was isolated using the TRIzol reagent (Invitrogen), then we used the reverse transcription kit (Qiagen) to synthesize cDNA. Gene expression was quantified by SYBR green real-time PCR on a Stratagene Mx3005 System (Agilent Technologies, Santa Clara, California, USA). The sequences of primer pairs are in Table S2 (Additional File 1). GAPDH was used as an internal control. Differences were calculated with the comparative threshold cycle (Ct) value.

### Cell viability assay

Fibroblasts were seeded at a density of 5 × 10^3^/well in 96-well flat bottom plates and were treated with different concentrations of iguratimod for 24, 72, and 120 h. The Cell Counting Kit-8 (Dojindo, Japan) was used according to the manufacturer’s instructions for the fibroblast viability assay.

### Western blot analyses

Fibroblasts were lysed in RIPA buffer (Thermo Fisher, USA). The lysate was electrophoresed on SDS-PAGE gel and then transferred to a polyvinylidene difluoride membrane. Proteins were detected with primary antibodies against EGR1 (Abcam, USA), collagen I (Abcam, USA), and β-Actin (Sigma-Aldrich, Germany), in addition to horseradish peroxidase (HRP)-conjugated secondary antibody (CST, USA). Finally, proteins were visualized using ChemiDoc Imaging Systems (bio-Rad, USA) and analyzed by Image J software (National Institute of Health, Bethesda, MD, USA).

### Assessment of the collagen

Collagen in fibroblast or tissue sections was detected by with Sirius red/fast green collagen staining kit (Chondrex, US). To evaluate skin fibrosis, the total collagen content of tissue samples was determined by hydroxyproline assays using the chloramines-T method. In addition, we measured the thickness of mouse dermis and counts of α-SMA positive myofibroblasts as previously described [18].

### Immunofluorescence and immunohistochemistry staining

Human dermal fibroblasts were fixed with 4% paraformaldehyde. 0.2% Triton X‑100 and 1% BSA were used sequentially for penetrating and blocking. fibroblasts were incubated with anti‑EGR1 (CST, USA) for 12 h at 4℃. Afterwards, fibroblasts were washed and incubated with secondary antibodies (Alexa Fluor 488 conjugated, CST, USA). Stress fibers were stained with rhodamine-conjugated phalloidin (Sigma-Aldrich, Germany). Nuclei were stained using DAPI (4′,6-diamidin-2-phenylindole, CST, USA). Images were captured using a Nikon Eclipse TE2000‑S fluorescence microscope.

For tissue staining, formalin-fixed, paraffin-embedded (FFPE) skin sections were deparaffinized and stained with anti-EGR1 (Abcam, USA) at anti-TGFβ (R&D system, USA), anti-α-SMA (R&D system, USA) antibody. HRP-conjugated secondary antibodies were used afterwards. For skin fibroblast counting, α-SMA/TGF-β/EGR1 positive fibroblast was counted as the mean value of two distinct sections for each mouse. Each section included five random HPF at 200 times magnification.

### Statistical analysis

Statistical analysis was performed with the GraphPad Prism 7 software. Statistical significance between two groups was calculated by the Mann–Whitney *U* test; for comparisons of more than two groups, one-way or RM one-way ANOVA with Bonferroni correction for multiple comparisons was used. *P* value < 0.05 was considered significant.

## Results

### EGR1 was predominantly overexpressed in non-immune cells of SSc patients

**Fig. 1:**
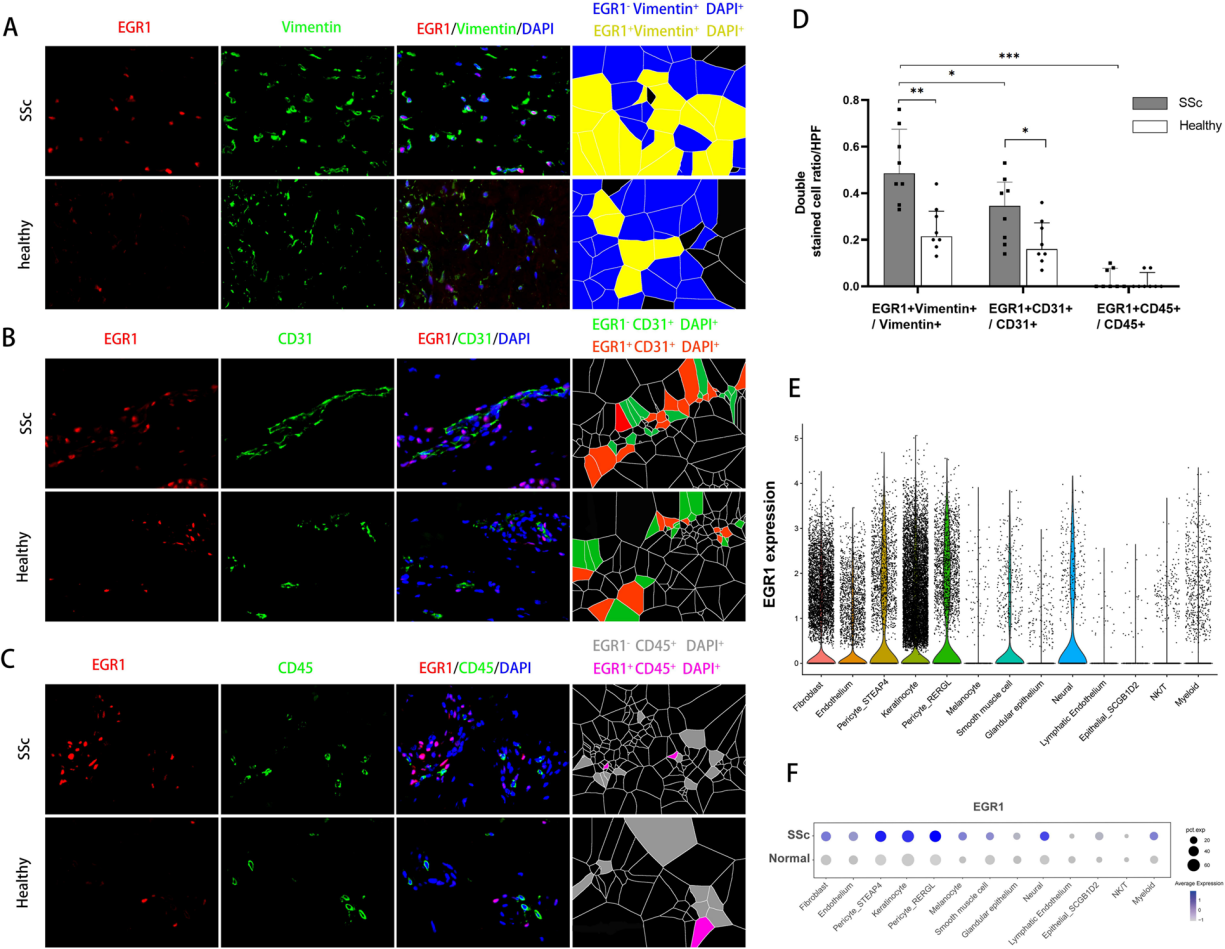
EGR1 increased in the dermis of SSc patients and was located predominantly in fibroblasts. **A**–**C** co-staining of EGR1 with vimentin, CD31, or CD45 in skin tissues from healthy donors (*n* = 8) and SSc patients (*n* = 8). **D** Quantification of EGR-positive cells’ ratio in fibroblasts (VIMENTIN-positive), endothelia (CD31-positive), or leukocytes (CD45-positive). One-way ANOVA with Bonferroni multiple comparisons test was used for statistical analysis. Data are represented as mean ± standard deviation. *, 0.05 > *P* > 0.01; **, 0.01 > *P* > 0.001; ***, *P* < 0.001. **E** EGR1 expression level in skin cell clusters of a validation single-cell sequencing dataset. **F** EGR1 expression between diffused SSc (*n* = 12) and normal control (*n* = 10) from the validation single-cell sequencing dataset

In skin sections from dcSSc patients and healthy volunteers, EGR1 was detected by immunofluorescence staining in VIMENTIN-positive fibroblasts (Fig. 1A) and CD31-positive endothelia (Fig. 1B) but could hardly be detected in CD45 positive leukocytes (Fig. 1C). Compared with healthy volunteers, both fibroblasts and endothelia showed increased EGR1 positive cell counts in the SSc dermis according to quantification (Fig. 1D). Consistently, the single-cell data GSE138669 (Fig. S1) indicated EGR1 was preferentially expressed in non-immune cells, such as fibroblast, keratinocyte, and pericyte (Fig. 1E), and was upregulated in dcSSc dermal cells (Fig. 1F).

### Iguratimod did not affect the proliferation of fibroblast

We performed a CCK8 assay to evaluate the effect of iguratimod on fibroblast proliferation. There were no inhibitory effects of iguratimod on fibroblasts for 1, 3, or 5 days at concentrations up to 100 μM (Fig. S2).

### Iguratimod inhibited EGR1 expression and neutralized EGR1 response genes in human dermal fibroblasts

**Fig. 2:**
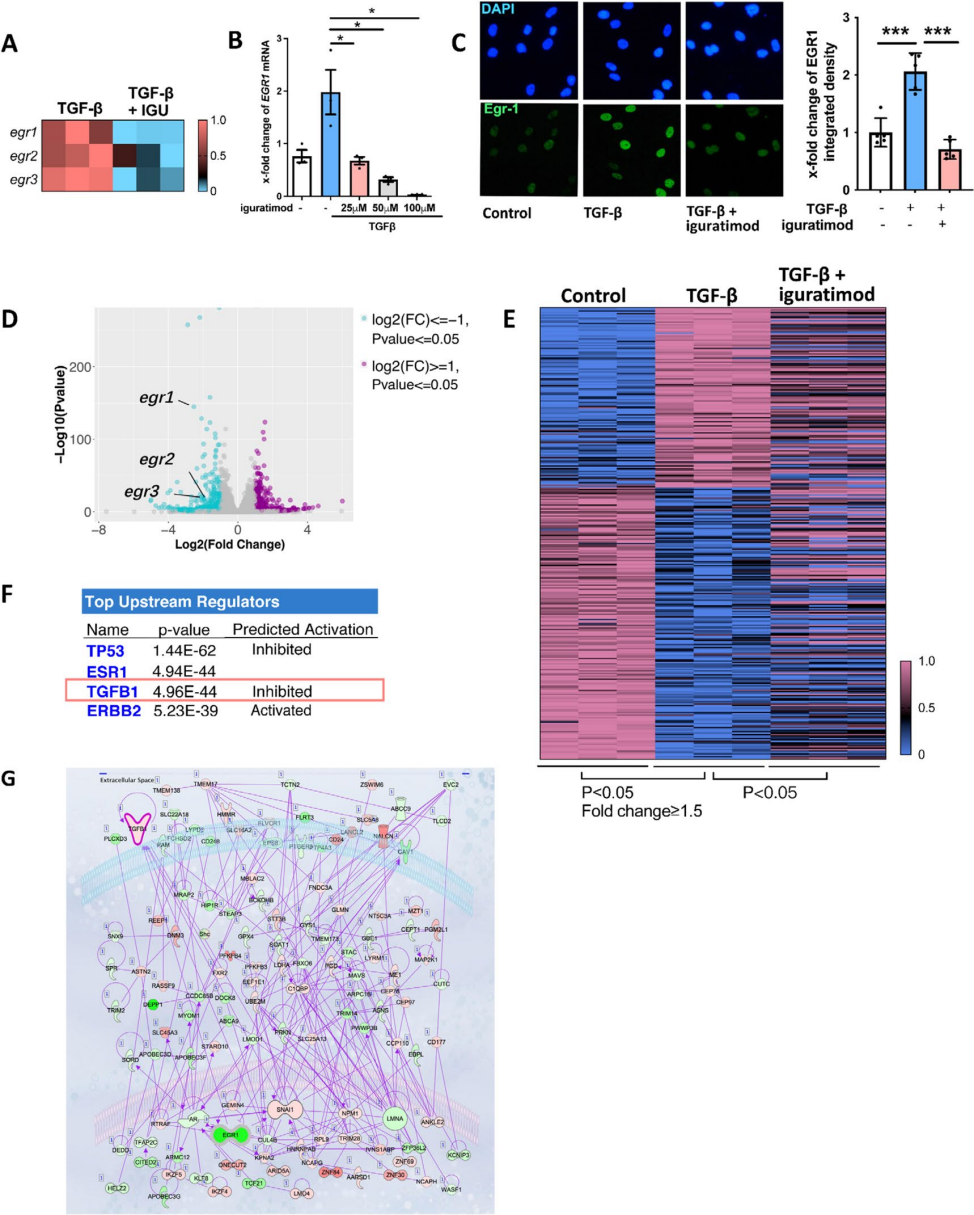
EGR1, as well as EGR1 response genes, were regulated by iguratimod in human dermal fibroblasts. Dermal fibroblasts from healthy donors were stimulated by TGFβ and treated with 0.1% DMSO or iguratimod in different concentrations. **A** Iguratimod suppressed the expression of the EGR family, particularly *EGR1*. Each cell represents normalized gene expression of an independent qPCR experiment. IGU, iguratimod **B** mRNA level of *EGR1* in human dermal fibroblasts treated with TGFβ and different concentrations of iguratimod *N* = 3 per group. *, 0.05 > *P* > 0.01; **, 0.01 > *P* > 0.001, one-way ANOVA with Bonferroni multiple comparisons test was used for statistical analysis. Data are represented as mean ± standard deviation. **C** immunofluorescence staining of EGR1 together with DAPI in human dermal fibroblasts treated with TGFβ or iguratimod (*N* = 5 per group). Representative image (400 ×) and quantification are included. One-way ANOVA with Bonferroni multiple comparisons test was used for statistical analysis, ***, *P* < 0.001. Data are represented as mean ± standard deviation. **D** Volcano plot showed the differentially expressed genes in TGF-β stimulated fibroblast, with or without iguratimod treatment. **E** Iguratimod largely neutralized the effects of TGF-β on EGR1 response genes. This heatmap shows 327 EGR1 response genes that could be significantly regulated by TGF-β. **F** List of the top upstream regulators calculated by IPA. **G** Component genes of the TGF-β pathway that changed with iguratimod treatment. Red represents up-regulation and green represents downregulation

Since multiple members of the EGR family are involved in fibrosis [19–22], we detected EGR family expression in iguratimod-treated human dermal fibroblasts through qPCR. The expression of *EGR1-3* was all reduced, whereas *EGR1* is the most significantly downregulated gene (Fig. 2A). Using concentration gradients of iguratimod, we found that iguratimod reduced *EGR1* expression in a dose-dependent manner (Fig. 2B). Immunofluorescence staining showed decreased EGR1 signal located in the nucleus, which supported the suppressive effect of EGR1 by iguratimod on protein level (Fig. 2C).

Further transcriptome profiling of fibroblast provided more information on EGR1 response genes. *EGR1* was one of the most significantly decreased genes with iguratimod treatment in fibroblasts (Fig. 2D). Then we identified EGR1 response genes that could be changed upon TGF-β stimulation. Among EGR1-response genes associated with fibrosis according to a previous report [12], we identified genes with fold change ≥ 1.5 and *P* value < 0.05 as differentially expressed genes in comparison of the TGF-β group versus normal control. A total of 327 EGR1 response genes were considered as significantly changed by TGF-β stimulation. We next compared groups with or without iguratimod treatment but both upon TGF-β stimulation and found that iguratimod could neutralize the effects of TGF-β on 232 out of 327 EGR1response genes (Fig. 2E).

we used IPA to further analyze the differentially expressed genes and identified that TGF-β was one of the top upstream regulators that were inhibited by iguratimod treatment (Fig. 2F), revealing that the TGF-β stimulation was largely invalidated by iguratimod. Furthermore, we visualized the differentially expressed components in the TGF-β downstream signaling pathway, most of which were downregulated upon iguratimod treatment with EGR1 reduced most (Fig. 2G). An additional protein–protein interaction (PPI) analysis using STRING database showed EGR1 in the central part of the iguratimod working network (Fig. S3).

### Iguratimod inhibited fibroblast activation and ECM synthesis

**Fig. 3:**
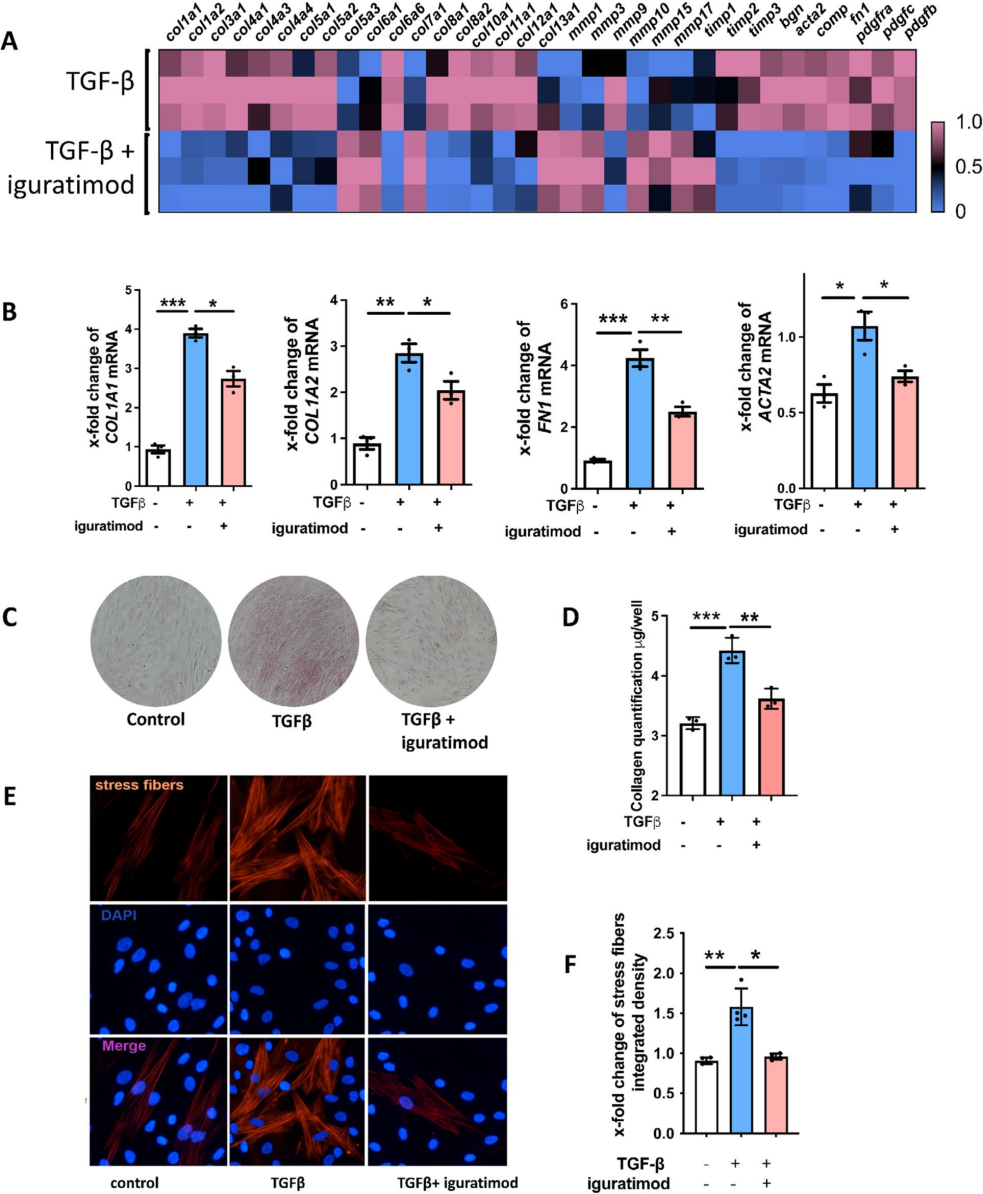
Iguratimod inhibited human skin fibroblast activation and function of extracellular matrix (ECM) synthesis. **A** Heatmap showed expression of collage, collage degradation system, and other major pro-fibrotic factors (*p*-value < 0.05). *N* = 3 per group. **B** mRNA expression of *COL1A1*, *COL1A2*, *FN1*, and *ACTA2* in human dermal fibroblasts upon TGFβ stimulation and iguratimod treatment. *N* = 3 per group. *, 0.05 > *P* > 0.01; **, 0.01 > *P* > 0.001, one-way ANOVA with Bonferroni multiple comparisons test was used for statistical analysis. Data are represented as mean ± standard deviation. **C** Representative images of Sirius red for intracellular collagen detection (magnification: 40 ×) **D** Quantification of collagen content by eluting the Sirius red dye and colorimetric measuring. *N* = 3 per group. **, 0.01 > *P* > 0.001, one-way ANOVA with Bonferroni multiple comparisons test was used for statistical analysis. Data are represented as mean ± standard deviation. **E** Representative image of stress fiber via rhodamine-phalloidin staining along with quantification (magnification: 400 × , *N* = 4 per group), *, 0.05 > *P* > 0.01, **, 0.01 > *P* > 0.001, one-way ANOVA with Bonferroni multiple comparisons test was used for statistical analysis. Data are represented as mean ± standard deviation

The differentially expressed genes of the RNASeq results between TGFβ-stimulated fibroblasts with or without iguratimod treatment showed that numerous profibrotic genes were downregulated, and most matrix metallopeptidase (MMP) genes involving collagen degradation were upregulated by iguratimod (Fig. 3A). The sequencing results were confirmed by qPCR in several representative profibrotic genes (Fig. 3B). Consistent with the RNA seq results, iguratimod inhibited TGFβ-induced myofibroblast differentiation with reduced release of collagen protein (Fig. 3C, D) and reduced formation of stress fibers (Fig. 3E, F) as compared to fibroblasts treated with vehicle.

### EGR1 was the key target in the anti-fibrotic process of iguratimod

**Fig. 4:**
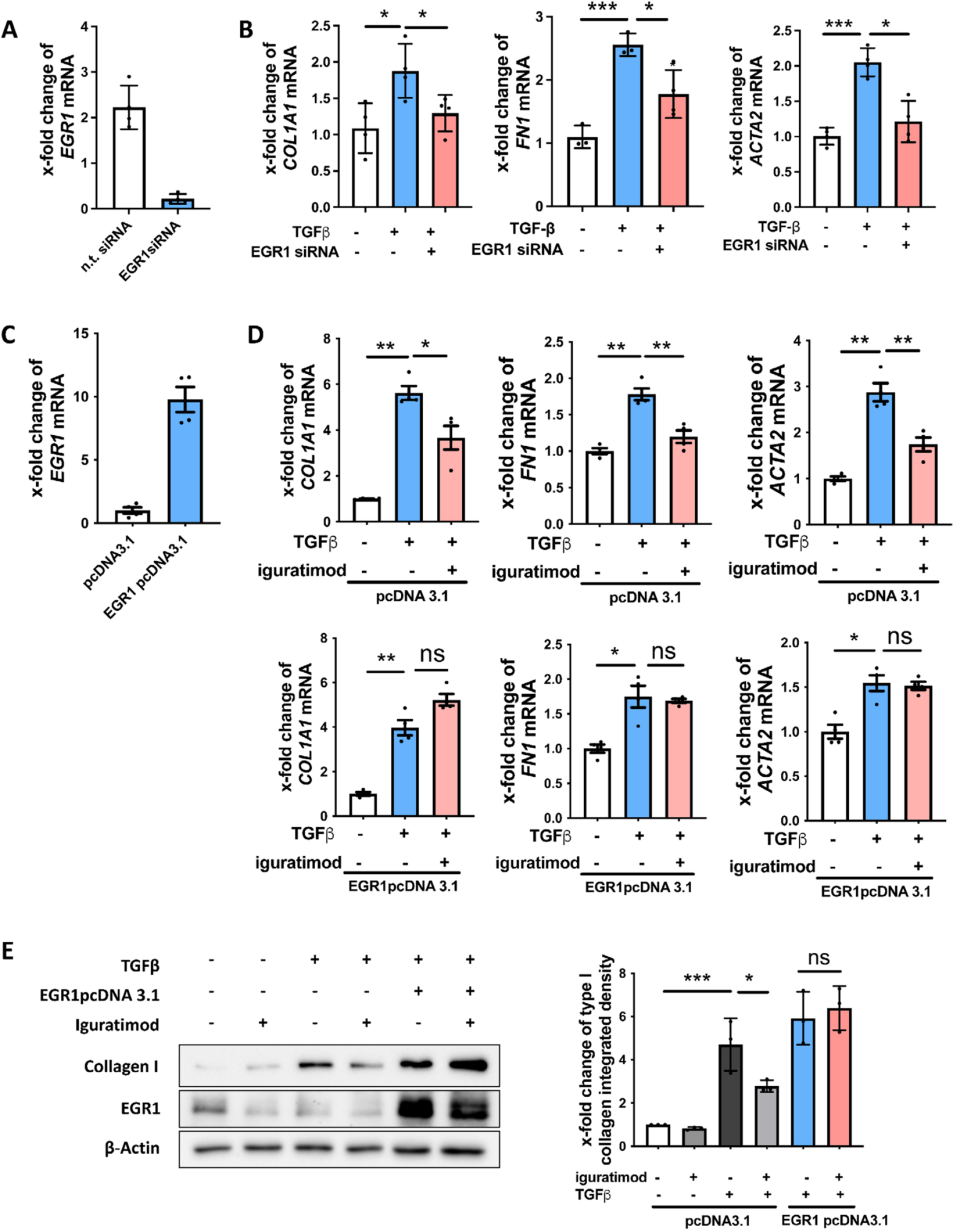
EGR1 was the key target for iguratimod to the inhibition of fibroblast function. **A**, **B** Knockdown of EGR1 inhibited fibroblast activation and ECM production. **A** Relative mRNA level of EGR1 in human dermal fibroblast transfected with n.t.siRNA or EGR1 siRNA. **B** Relative mRNA level of *COL1A1*, *FN1*, and *ACTA2* in human dermal fibroblast transfected with n.t.siRNA or EGR1 siRNA and treated with or without TGFβ. *N* = 4 per group. **C**–**E** EGR1 overexpression neutralized the inhibition of fibroblast activation and ECM production treated with iguratimod. **C** Relative mRNA level of EGR1 in human dermal fibroblast transfected with pcDNA3.1or EGR1 pcDNA3.1. **D** Relative mRNA level of *COL1A1, FN1*, and *ACTA2* in human dermal fibroblast transfected with pcDNA3.1or EGR1 pcDNA3.1 and treated with iguratimod or TGFβ. *N* = 4 per group. **E** Protein level of EGR1 and collagen I in human dermal fibroblast transfected with pcDNA3.1or EGR1 pcDNA3.1 and treated with iguratimod or TGFβ. The representative image and quantification were included *N* = 3 per group. *, 0.05 > *P* > 0.01; **, 0.01 > *P* > 0.001; ***, *P* < 0.001. Mann–Whitney testing was used for statistical analyses in **A** and **C**. One-way ANOVA with Bonferroni multiple comparisons test was used in **B**, **D**, and **E**. Data are represented as mean ± standard deviation

To confirm the role of EGR1 in fibroblast activation and function, we knocked down EGR1 in fibroblasts by transfecting EGR1 siRNA (Fig. 4A). Consistent with iguratimod, EGR1-targeted siRNA inhibited the expression of *COL1A1*, *COL1A2*, *FN1*, and *ACTA2* in human dermal fibroblasts compared with non-target siRNA (Fig. 4B, S4).

Furthermore, we performed EGR1 overexpression in human dermal fibroblasts by transfecting plasmid cDNA3.1 encoding EGR1 open reading frame (Fig. 4C). As expected, the anti-fibrotic effect of iguratimod was abrogated by EGR1 overexpression on both mRNA and protein levels (Fig. 4D, E).

### Iguratimod ameliorated pre-established bleomycin-induced dermal fibrosis

**Fig. 5:**
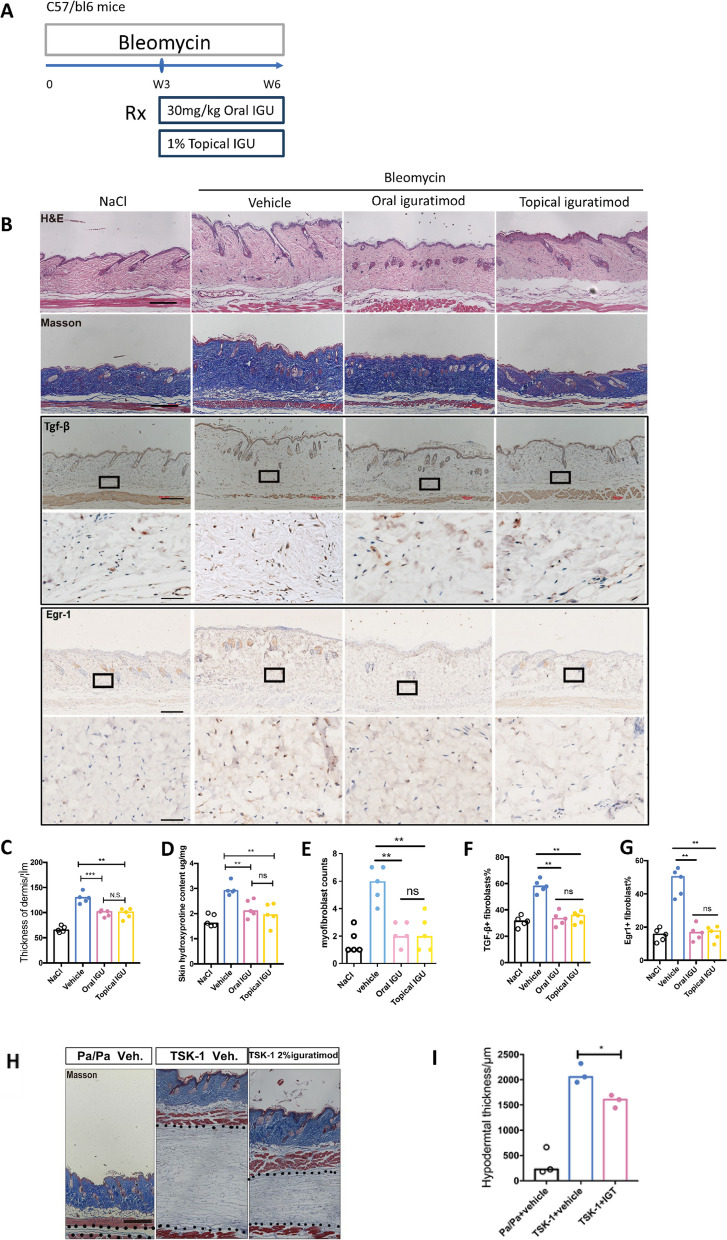
Effects of iguratimod in experimental dermal fibrosis murine model. **A**–**G** Effects of iguratimod in bleomycin-induced dermal fibrosis murine model. Male C57/BL6 mice were subcutaneously injected with bleomycin for 6 weeks. Since the fourth week, oral (30 mg/kg) or topical (2%) iguratimod was administrated every day with bleomycin **A**. **B** Representative H&E, Masson, and Tgf-β/Egr1 staining of the skin. In lines 1–3 and 5, scale bars = 500 μm; in lines 4 and 6, scale bar = 250 μm. **C** Measurement of dermis thickness. **D** Quantification of hydroxyproline content. **E** α-SMA + myofibroblast counts in 200 × field. **F** and **G** The proportion of Tgf-β ( +) and Egr1 ( +) fibroblasts in each 200 × field. **H**–**I** Effects of Iguratimod in tight skin-1 (Tsk-1) mice. Tsk-1 mice were treated with 2% iguratimod topically twice a day for 42 days. **H** Representative images of Masson staining. Dot lines indicated hypodermal layers beneath the panniculus carnosus. Bar, 500 μm. **I** Hypodermal thickness. Bars represent median. IGU, iguratimod. *, 0.05 > *P* > 0.01; **, 0.01 > *P* > 0.001; ***, *P* < 0.001, one-way ANOVA with Bonferroni multiple comparisons test was used for statistical analysis, data are represented as median (**B**–**G**, **I**)

Oral or topical iguratimod was given to male C57/bl6 mice with pre-established skin fibrosis induced by bleomycin (Fig. 5A). After a 3-week treatment, both administrations of iguratimod significantly decreased the dermis thickness, as well as collagen deposition and hydroxyproline content (Fig. 5B–D). α-SMA positive myofibroblast density was decreased remarkably (Fig. 5E). The therapeutic effect was comparable between topical and oral iguratimod. No observable adverse effect was present.

We further detected Egr1 and Tgf-β in fibrotic skin tissue through immunohistochemistry. The proportions of Egr1 and TGF-β positive fibroblasts were simultaneously decreased in iguratimod-treated groups. No statistically significant difference was found between systemic and topical treatment (Fig. 5B, F, and G).

### Iguratimod reduced spontaneously hypodermis fibrosis in Tsk-1 mice

We used Tsk-1 mice which is a spontaneously onset SSc mouse model different from bleomycin-induced skin fibrosis, with hyperplasia of the hypodermis and less inflammation Tsk-1 mice were treated with 1% iguratimod DMSO solution topically twice a day for 6 weeks. Again, the treatment reduced hypodermal thickening as well as the collagen deposition in skin tissues compared with controls of DMSO (Fig. 5H, I, and S5).

### Iguratimod attenuated fibrosis of skin grafts from SSc patients

**Fig. 6:**
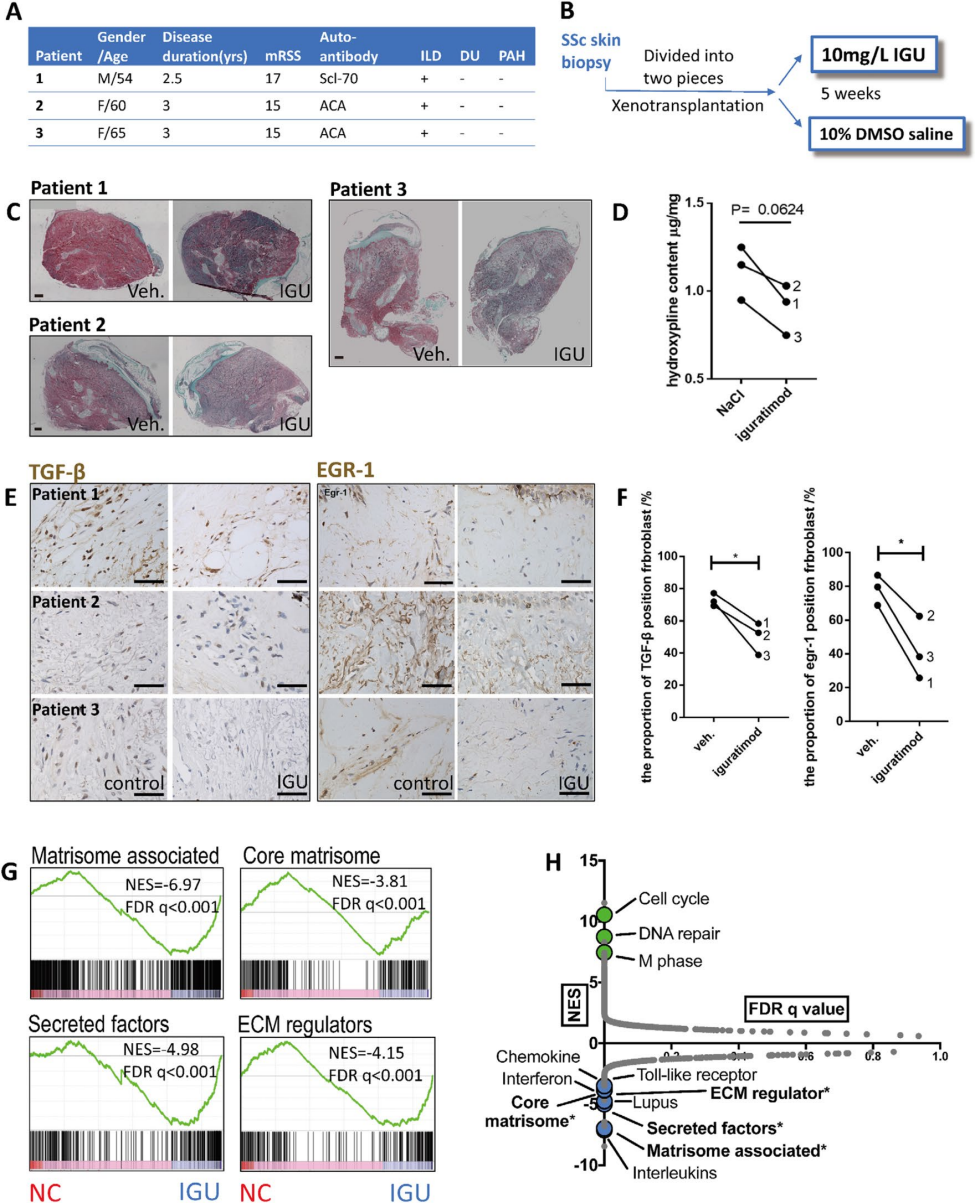
Effects of iguratimod in skin grafts from naïve SSc patients. **A** Clinical characteristics of the three SSc patients. All patients were treatment naïve. Disease duration was measured from the onset of the first non-Raynaud symptoms attributable to SSc. Interstitial lung disease was diagnosed by high-resolution CT. Pulmonary arterial hypertension was screened by ultrasound. (**B** Each lesional skin tissue from the patients was transplanted subcutaneously into two irradiated nude mice. The grafts were injected with 0.1 mg/ml iguratimod or vehicle every day for 5 weeks. **C** Sirius red staining for each graft. The red parts represent collagen and the green parts represent total protein. Scale bars = 1 mm. **D** The collagen content of each skin tissue was quantified by eluting the dye and colorimetric assay. **E** Representative images of EGR1 and TGFβ staining. Scale bar = 125 μm. **F** The proportion of TGFβ ( +) and EGR1 ( +) fibroblasts in each mm^2^. **G** GSEA of representative gene ensembles associated with ECM production. Data from RNA-Seq of all skin grafts treated with or without iguratimod. **H** Plot of FDR versus the NES based upon GSEA of each possible gene ensemble. * represents ECM production associated gene ensembles. **D** and **F** Mann–Whitney *U* test was used for statistical analyses. *, 0.05 > *P* > 0.01. mRSS, the modified Rodnan skin score; ILD, interstitial lung disease; DU, digital ulcer; PAH, pulmonary arterial hypertension; ACA, anti-Centromere antibody; IGU, iguratimod; NES, normalized enrichment score; FDR, false discovery rate

To evaluate the possibility of iguratimod working on SSc patients in clinical practice, we collected skin biopsies from three early SSc patients. All of them had symptoms other than the Reynaud phenomenon no more than 3 years and had never received immunosuppressants, anti-fibrotic, or steroid treatment. Detailed clinical information of the patients is shown in Fig. 6A.

Three grafts were divided equally, transplanted into two irradiated nude mice, and treated with iguratimod or vehicle solution respectively by subcutaneous injection for 5 weeks (Fig. 6B). Sirius red staining of skin sections showed a visible reduction of collagen deposition upon iguratimod treatment (Fig. 6C). Indeed, quantification of skin hydroxyproline content showed a decreased trend in iguratimod-treated skin tissues (Fig. 6D). Similarly, staining of EGR1 and TGF-β showed a double decrease (Fig. 6E), with statistical significance on EGR1 or TGF-β positive fibroblast proportion (Fig. 6F).

In further RNA sequencing of the skin grafts, the ensemble of genes encoding extracellular matrix and ECM-associated proteins, which was termed as matrisome, was the most enriched category among the GSEA result. A comprehensive decrease was identified in genes both encoding core matrisome proteins (including ECM glycoproteins, collagens, and proteoglycans) and encoding matrisome-associated proteins (including ECM-affiliated proteins, ECM regulators, and secreted factors) (Fig. 6G, Fig. S6). In these ensembles, there were pro-fibrotic cytokine genes such as *TGFB1* and *FGFs*, and *WNTs*; ECM degradation enzymes genes such *MMPs* and *CTSs*; ECM-associated chemokines such as *CCL13* and *CCL18*; and notably, a remarkable amount of glycoprotein/ proteoglycan genes such as *LRG1*, *PRG4*, *HAPLN1*, and *PODNL1*. On the other side, the GSEA showed some categories related to the innate immune response in the top downregulated gene sets as well, such as interleukin, chemokine, toll-like receptor, and the complement system (Fig. 6H, Fig. S6), which supported the role of iguratimod as an anti-rheumatic drug that was found previously.

## Discussion

This is the first study to investigate the EGR1 expression in different cell types in SSc skin. We show this phenomenon not only in our data but at a single-cell level via a validation dataset. Interestingly, although EGR1 has been reported as ubiquitously expressed before, this gene was predominantly expressed in non-immune cells but not in leukocytes from the SSc dermis, especially highly expressed in fibroblasts. Therefore, targeting EGR1 expression in SSc patients would be a reasonable anti-fibrotic approach rather than anti-inflammation.

On top of that, we demonstrated for the first time that for iguratimod, EGR1 was probably the key against fibrosis and to regulating TGF-β signaling. As a small-molecular compound, iguratimod is recognized to have multiple effects, such as inhibiting NF-κB translocation [23] and dampening IL-17 signaling by targeting Act1 [24]. We are the first group using high-throughput approaches to identify the mechanism of iguratimod and find EGR1 as one of the most downregulated genes [16]. In this work, we confirm this result among EGR family genes and the dose-dependent inhibition of EGR1 expression. More importantly, EGR1 overexpression neutralized the anti-fibrotic effect of iguratimod. All these support that EGR1 inhibition could be a major mechanism of iguratimod to the anti-fibrotic effect of iguratimod.

Other evidence in this study supported a parallel anti-fibrotic effect of iguratimod probably rather than merely the consequence of suppressed inflammation [25]. For in vivo evidence, we selected Tsk-1 mice to demonstrate the anti-fibrotic effect of iguratimod, a model characterized by primary activation of fibroblast without obvious inflammation [26]. For in vitro evidence, iguratimod not only directly suppressed the function of skin fibroblast in producing collagen but also regulated a series of genes pivotal for fibrosis. The expression of PDGFs, a major cytokine family involved in SSc, was downregulated in our study. The MMP/TIMP system was also involved, of which MMPs, functioning in collage degradation, and TIMPs were the inhibitors of MMPs. As an agent with bone protective effect for RA patients, iguratimod has been long noticed to regulate the MMP family. However, the regulation of MMPs by iguratimod differed with different conditions. For arthritis, iguratimod has been demonstrated to inhibit serum MMP-1 and MMP-3 of RA patients [27]; while in bleomycin-induced interstitial lung disease, iguratimod increased the level of MMP-9 of lung tissues [28]. Our study provided a more comprehensive view of human dermal fibroblast: most MMP genes’ expression, including *MMP1* and *MMP3*, were increased, with only *MMP9* slightly decreased while most TIMP genes’ expression decreased simultaneously (Fig. 3A).

Mechanically, both anti-fibrotic and immune regulatory treatments work for SSc, which has been demonstrated in clinical trials [29–31]. Furthermore, subgroup analysis shows these two can work in synergy [29]. Our study shows that iguratimod is an agent with the dual effect of anti-fibrotic and immune regulation. Besides anti-fibrotic, iguratimod decreases interleukin gene sets that were enriched by GSEA in SSc skin xenograft, in consist with previous findings of iguratimod inhibiting IL-17 producing, macrophage migration inhibitory factor (MIF), IL-6 and IL-1β, and suppress of NF-κB activation [24, 32–34]. Adaptive immune genes are enriched on the top of the gene-set list by GSEA as well, supporting the regulatory effect of iguratimod against auto-reactive T cells [35] and B cells [16, 36]. Our study implicates the potentially individualized application for autoimmune disease patients with fibrotic complications.

For clinical importance, though a remarkable advantage has been achieved in SSc treatment, 4.06% of standard mortality ratio and up to 22–26 years of life loss [37] suffered from the patients are still urgently asking for new efficient treatment. Unfortunately, only a few agents have succeeded in phase III clinical trials for SSc so far, especially for skin fibrosis. Under this circumstance, drug repurposing is a feasible and effective approach to meeting the clinical request. In our study, we not only show the efficacy of iguratimod in cultured fibroblast and mice but show the remarkably decreased collagen deposition in skin xenografts of naïve SSc patients, which strengthens the clinical implications of this work. Both oral and topical administration shown in this study extended the possible application scenario of iguratimod.

## Conclusion

In summary, as a reagent effectively interrupting EGR1 expression, iguratimod is a promising disease-modifying drug with an anti-fibrotic effect. It is reasonable to further investigate the efficacy of iguratimod for SSc in the context of clinical practice, as well as other autoimmune diseases with fibrotic complications.

### Supplementary Information

**Additional file 1: Figure S1.** Single-cell clustering (A) and marker genes (B) of skin tissue of diffused SSc (*n*=12) and normal control (*n*=10) from GSE138669. **Figure S2.** The concentration of iguratimod in this study did not affect fibroblast viability. Normal human dermal fibroblasts treated with different concentrations of iguratimod for 1 day, 3 days and 5 days to measure the cell viability by CCK-8 test. **, 0,001 < *P* <  0.01, one-way ANOVA with Bonferroni multiple comparisons test was used for statistical analysis. **Figure S3.** Gene interaction analysis on the top 50 regulated genes in TGF-β treated fibroblast with or without iguratimod treatment, using STRING database. EGR1 was in the center of the network. **Figure S4.** The effect of EGR1 knockout to the function of collagen synthesis in human dermal fibroblast Protein level of collagen I in human dermal fibroblast transfected with EGR1siRNA and n.t. siRNA. The representative image (A) and quantification (B) were included. *N* = 4 per group. Mann-Whitney testing was used for statistical analyses *, 0.05 > *P* > 0.01. **Figure S5.** Effects of iguratimod in experimental dermal fibrosis murine model. Protein level of collagen 1 in skin tissues from wildtype control mice and Tsk-1 mice treated with 2% iguratimod or solvent. The representative image and quantification were included *N* = 3 per group. *, 0.05 > *P* > 0.01; **, 0.01 > *P* > 0.001; ***, *P* < 0.001. One-way ANOVA with Bonferroni multiple comparisons test was used for statistical analysis. Data are represented as mean ± standard deviation. **Figure S6.** Representative gene ensembles of GSEA from RNA-seq of SSc skin xenografts. (A) other ECM production-related, (B) immune or inflammation related and (C) cell proliferation related gene ensembles. NES, normalized enrichment score; FDR, false discovery rate. **Table S1.** Characteristics of SSc patients with skin biopsies for EGR1/VIM immunofluorescence staining^a^. **Table S2.** Primer sequences for qPCR.

## Data Availability

Data are available in public, open-access repositories. The datasets for RNA-seq analyzed during the current study are available on GEO/SRA (GSE146478 and PRJNA606948).
