# Correction: Iguratimod attenuated fbrosis in systemic sclerosis via targeting early growth response 1 expression

**DOI:** 10.1186/s13075-024-03268-y

**Published:** 2024-01-22

**Authors:** Lichong Shen, Hanlin Yin, Li Sun, Zhiliang Zhang, Yuyang Jin, Shan Cao, Qiong Fu, Chaofan Fan, Chunde Bao, Liangjing Lu, Yifan Zhan, Xiaojiang Xu, Xiaoxiang Chen, Qingran Yan

**Affiliations:** 1 Department of Rheumatology, Ren Ji Hospital, Shanghai Jiao Tong University School of Medicine, Shanghai, 200001 China; 2 Department of Rheumatology and Immunology, The First Affiliated Hospital of Wenzhou Medical University, Wenzhou, 325000 China; 3 Department of Plastic Surgery, Ren Ji Hospital, Shanghai Jiao Tong University School of Medicine, Shanghai, 200001 China; 4 Department of Drug Discovery, Shanghai Huaota Biopharm, Shanghai, 201203 China; 5 Department of Pathology and Laboratory Medicine, Tulane University School of Medicine, New Orleans, LA USA; 6 Department of Rheumatology, Nantong First People’s Hospital, Affiliated Hospital 2 of Nantong Universuty, Nantong Hospital of Renji Hospital Affiliated to Shanghai Jiao Tong Universuty School of Medicine, Nantong, 226006 China

**Correction: Arthritis Res Ther 25, 151 (2023)**

**https://doi.org/10.1186/s13075-023-03135-2**

Following publication of the original article [1], the authors reported an error found in the Funding section, the grant number for Shanghai Municipal Commission of Health and Family Planning is incorrect. This should be corrected from “20204Y0088” to “**20204Y0090**”.

The original article [1] has been updated.
