# Supplementary material for: Iguratimod attenuated fibrosis in systemic sclerosis via targeting early growth response 1 expression
Source: Arthritis Res Ther. 2023 Aug 18;25:151. doi: 10.1186/s13075-023-03135-2 (PMC10439582; doi:10.1186/s13075-023-03135-2)
Supplement: Supplementary file 1 — Additional file 1: Figure S1. Single-cell clustering (A) and marker genes (B) of skin tissue of diffused SSc (n=12) and normal control (n=10) from GSE138669. Figure S2. The concentration of iguratimod in this study did not affect fibroblast viability. Normal human dermal fibroblasts treated with different concentrations of iguratimod for 1 day, 3 days and 5 days to measure the cell viability by CCK-8 test. **, 0,001 < P < 0.01, one-way ANOVA with Bonferroni multiple comparisons test was used for statistical analysis. Figure S3. Gene interaction analysis on the top 50 regulated genes in TGF-β treated fibroblast with or without iguratimod treatment, using STRING database. EGR1 was in the center of the network. Figure S4. The effect of EGR1 knockout to the function of collagen synthesis in human dermal fibroblast Protein level of collagen I in human dermal fibroblast transfected with EGR1siRNA and n.t. siRNA. The representative image (A) and quantification (B) were included. N = 4 per group. Mann-Whitney testing was used for statistical analyses *, 0.05 > P > 0.01. Figure S5. Effects of iguratimod in experimental dermal fibrosis murine model. Protein level of collagen 1 in skin tissues from wildtype control mice and Tsk-1 mice treated with 2% iguratimod or solvent. The representative image and quantification were included N = 3 per group. *, 0.05 > P > 0.01; **, 0.01 > P > 0.001; ***, P < 0.001. One-way ANOVA with Bonferroni multiple comparisons test was used for statistical analysis. Data are represented as mean ± standard deviation. Figure S6. Representative gene ensembles of GSEA from RNA-seq of SSc skin xenografts. (A) other ECM production-related, (B) immune or inflammation related and (C) cell proliferation related gene ensembles. NES, normalized enrichment score; FDR, false discovery rate. Table S1. Characteristics of SSc patients with skin biopsies for EGR1/VIM immunofluorescence staininga. Table S2. Primer sequences for qPCR. [file 13075_2023_3135_MOESM1_ESM.docx]

**Supplementary materials**

**Iguratimod attenuated fibrosis in systemic sclerosis via targeting early growth response 1 expression**

Lichong Shen^1, 6^, Hanlin Yin^1, 6^, Li Sun^2, 6^, Zhiliang Zhang^3^, Yuyang Jin^1^, Shan Cao^1^, Qiong Fu^1^, Chaofan Fan^1^, Chunde Bao^1^, Liangjing Lu^1^, Yifan Zhan^4^, Xiaojiang Xu^5,7^, Xiaoxiang Chen^1,7^, and Qingran Yan^1,7^

1. Department rheumatology, Ren Ji hospital, Shanghai Jiao Tong University school of medicine. Shanghai 200001, China.

2. Department of Rheumatology and Immunology, The First Affiliated Hospital of Wenzhou Medical University, Wenzhou 325000, China

3. Department plastic surgery, Ren Ji hospital, Shanghai Jiao Tong University school of medicine. Shanghai 200001, China.

4. Department of Drug Discovery, Shanghai Huaota Biopharm. Shanghai 201203, China.

5. Department of Pathology and Laboratory Medicine, Tulane University School of Medicine, New Orleans, LA, United States.

6. These authors contributed equally.

7. These authors contributed equally.

**Correspondence to:** Prof. Xiaoxiang Chen, xiaoxiang0721@126.com, Prof. Xiaojiang Xu, xxu17@tulane.edu and Dr. Qingran Yan, yanqingran@renji.com.

**Figure S1.** Single-cell clustering (A) and marker genes (B) of skin tissue of diffused SSc (n=12) and normal control (n=10) from GSE138669.


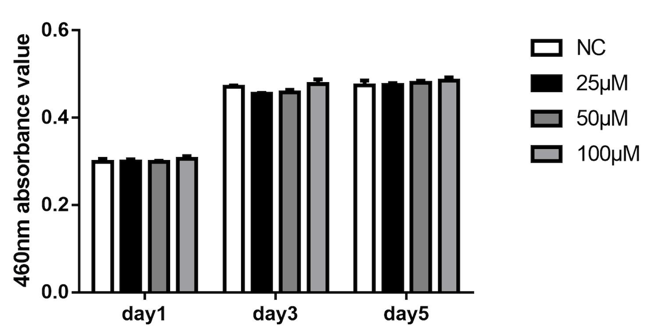


**Figure S2. The concentration of iguratimod in this study did not affect fibroblast viability.** Normal human dermal fibroblasts treated with different concentrations of iguratimod for 1 day, 3 days and 5 days to measure the cell viability by CCK-8 test. **, 0,001 < P <  0.01, one-way ANOVA with Bonferroni multiple comparisons test was used for statistical analysis.


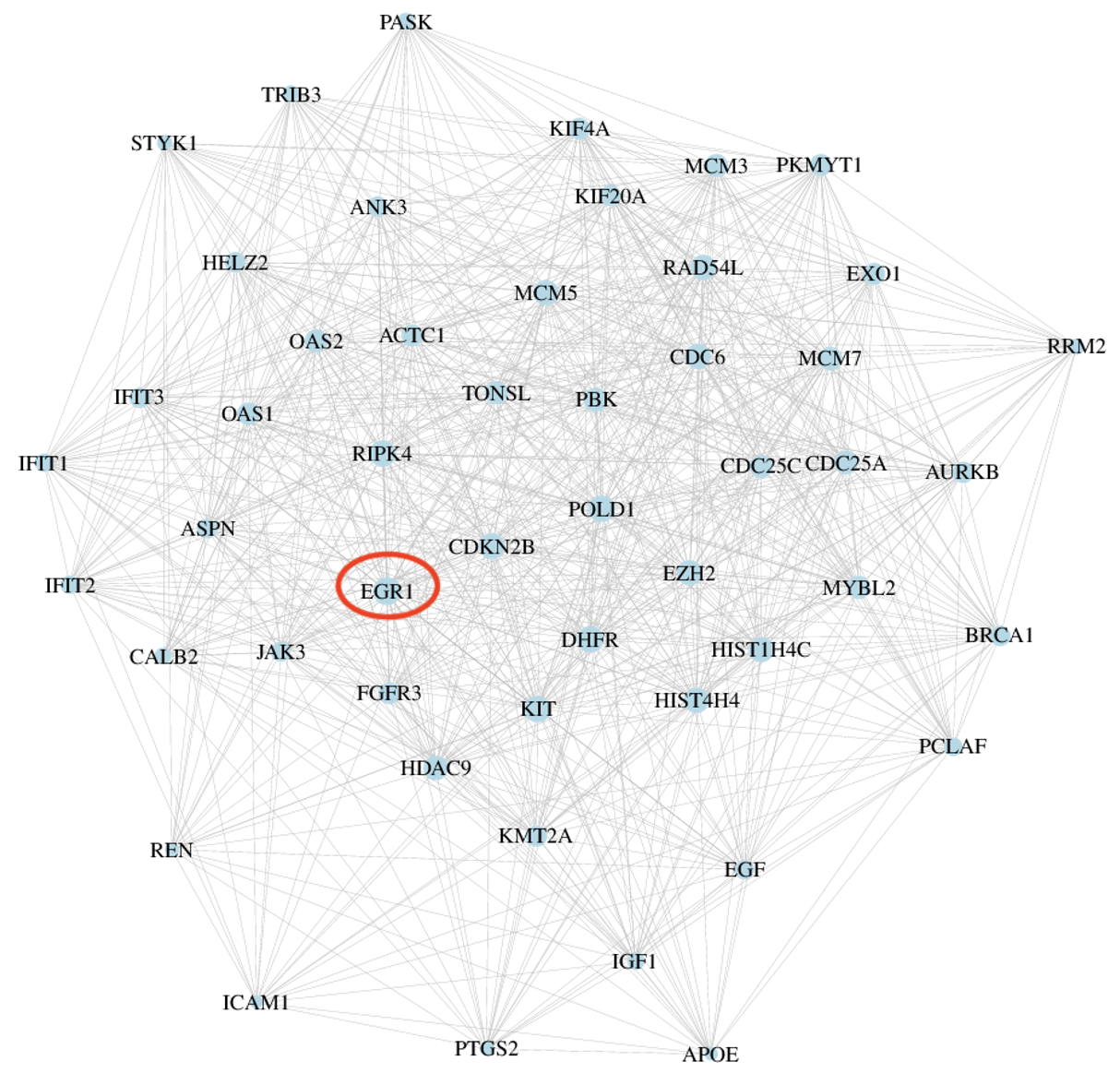


**Figure S3.** Gene interaction analysis on the top 50 regulated genes in TGF-β treated fibroblast with or without IGT treatment, using STRING database. Egr1 was in the center of the network.

**A B**


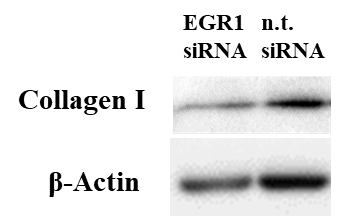


**Figure S4 The effect of EGR1 knockout to the function of collagen synthesis in human dermal fibroblast** Protein level of collagen I in human dermal fibroblast transfected with EGR1siRNA and n.t. siRNA. The representative image (A) and quantification (B) were included. N = 4 per group. *, 0.05 > P > 0.01.

**A**


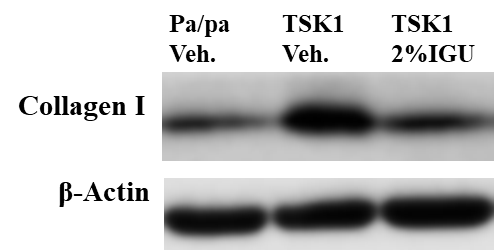


**B**

**
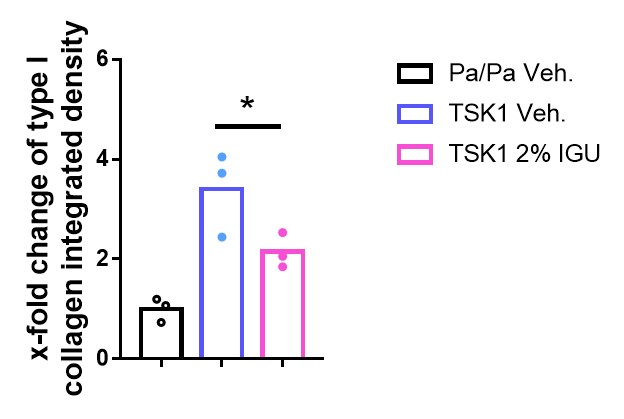
**

**Figure S5 Effects of iguratimod in experimental dermal fibrosis murine model.** Protein level of collagen 1 in skin tissues from wildtype control mice and TSK1 mice treated with 2% iguratimod or solvent. The representative image and quantification were included N = 3 per group. *, 0.05 > P > 0.01; **, 0.01 > P > 0.001; ***, P < 0.001. One-way ANOVA with Bonferroni multiple comparisons test was used for statistical analysis. Data are represented as mean ± standard deviation.


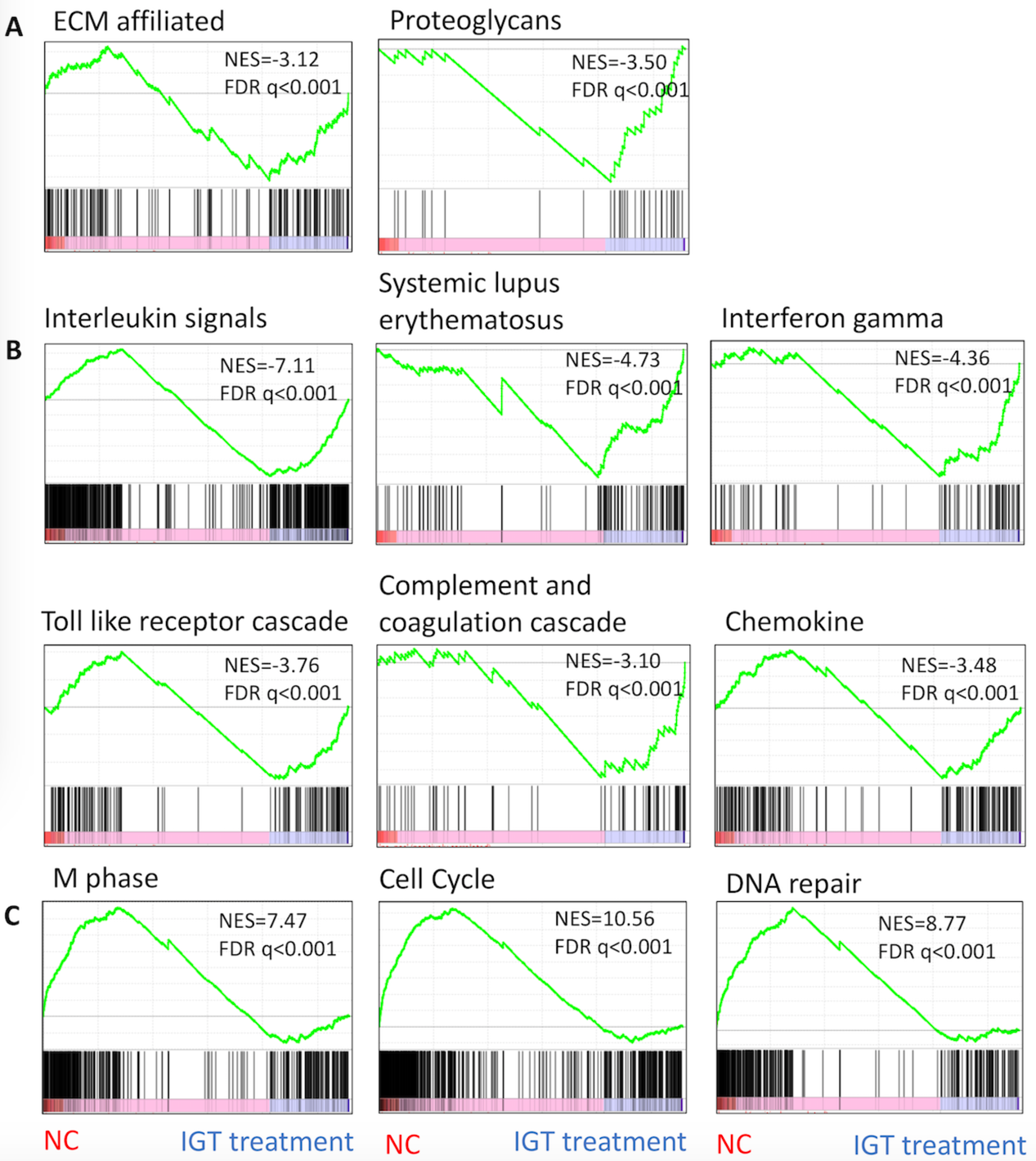


**Figure S6. Representative gene ensembles of GSEA from RNA-seq of SSc skin xenografts.** (A) other ECM production-related, (B) immune or inflammation related and (C) cell proliferation related gene ensembles. NES, normalized enrichment score; FDR, false discovery rate.

**Table S1. Characteristics of SSc patients with Egr1 skin staining^a^.**

| **Patient No.** | **Age** | **Sex** | **Duration^b^ (m)** | **Autoantibody** | **mRSS** | **ILD^c^** | **PAH^d^** | **DU^e^** | **Proteinuria** | **synovitis/myositis** | **Recent^f^ IS** | **Recent^f^ anti-fibrotic agents** |
| --- | --- | --- | --- | --- | --- | --- | --- | --- | --- | --- | --- | --- |
| 1 | 57 | M | 6 | ATA | 30 | Yes | No | Yes | Yes | No | TOF | / |
| 2 | 74 | F | 120 | ATA | 16 | Yes | Yes | Yes | No | No | / | / |
| 3 | 56 | F | 18 | ATA | 10 | Yes | No | No | No | No | / | / |
| 4 | 43 | F | 9 | ANA only | 10 | Yes | No | No | No | No | CYC | / |
| 5 | 28 | F | 55 | nRNP | 8 | No | No | Yes | No | No | MMF | / |
| 6 | 68 | F | 48 | ATA | 10 | Yes | Yes | No | Yes | No | / | Pirfenidone |
| 7 | 63 | F | 5 | nRNP | 28 | Yes | Yes | No | No | Yes | / | / |
| 8 | 47 | F | 18 | U3RNP | 9 | Yes | No | Yes | No | Yes | MMF | Nintedanib |

a. All characteristics were counted at the time of the skin biopsy.

b. Since the first non-Reynaud phenomenon.

c. X-ray or HRCT confirmed.

d. Right heart catheter confirmed or echocardiography with peak tricuspid regurgitation velocity >3.4 m/s.

e. Currently present or previous DUs were recorded as "yes"; "No" represented DU never happened.

f. Any eligible treatments applied within three months before the skin biopsy.

ATA, anti-topoisomerase I antibody; ANA, anti-nuclear antibody; mRSS, the modified Rodnan skin score; ILD, interstitial lung disease; PAH, pulmonary arterial hypertension; DU, digital ulcer; IS, immunosuppressant agents; TOF, tofacitinib; CYC, cyclophosphamide; MMF, mycophenolate mofetil.

**Table S2. Primer sequences for qPCR**

| gene | forward | reverse |
| --- | --- | --- |
| *GAPDH* | 5’-GAAGGTGAAGGTCGGAGTC-3’ | 5’-GAAGATGGTGATGGGATTTC-3’ |
| *EGR1* | 5’-TGCGGCAGAAGGACAAGAAAGC-3’ | 5’-TGAGGAAGGGAAGCTGCTGACC-3’ |
| *COL1A1* | 5’-GGACACAGAGGTTTCAGTGGT-3’ | 5’-GCACCATCATTTCCACGAGC-3’ |
| *COL1A2* | 5’- GGCTGAGAGGTAGTCCTGGT-3’ | 5’-GGCGACCAGCATCTCCATTA-3’ |
| *FN1* | 5’-GCCTGGTACAGAATATGTAGTG-3’ | 5’- ATCCCAGCTGATCAGTAGGCTGGTG-3’ |
| *ACTA2* | 5’-TATCCCCGGGACTAAGACGG-3’ | 5’- CACCATCACCCCCTGATGTC-3’ |
